# Automated recognition of emotional states of horses from facial expressions

**DOI:** 10.1371/journal.pone.0302893

**Published:** 2024-07-15

**Authors:** Marcelo Feighelstein, Claire Riccie-Bonot, Hana Hasan, Hallel Weinberg, Tidhar Rettig, Maya Segal, Tomer Distelfeld, Ilan Shimshoni, Daniel S. Mills, Anna Zamansky

**Affiliations:** 1 Information Systems Department, University of Haifa, Haifa, Israel; 2 Computer Science Department, University of Haifa, Haifa, Israel; 3 Department of Life Sciences, Joseph Banks Laboratories, University of Lincoln, Lincoln, United Kingdom; 4 Faculty of Electrical Engineering, Technion, Israel Institute of Technology, Haifa, Israel; Eskisehir Osmangazi University: Eskisehir Osmangazi Universitesi, TURKEY

## Abstract

Animal affective computing is an emerging new field, which has so far mainly focused on pain, while other emotional states remain uncharted territories, especially in horses. This study is the first to develop AI models to automatically recognize horse emotional states from facial expressions using data collected in a controlled experiment. We explore two types of pipelines: a deep learning one which takes as input video footage, and a machine learning one which takes as input EquiFACS annotations. The former outperforms the latter, with 76% accuracy in separating between four emotional states: baseline, positive anticipation, disappointment and frustration. Anticipation and frustration were difficult to separate, with only 61% accuracy.

## Introduction

The prevailing consensus now acknowledges that animals experience not only negative emotions such as fear and distress [1], but also positive emotional states [2]. While the historical focus of animal welfare science centered on pain and suffering, a recent notable shift in perspective encompasses a broader evaluation of their overall quality of life [3]. This shift leads to increased interest also in animal emotion research, and specifically positive emotional states [4, 5].

Facial expressions are an important information channel for affective states in animals. Charles Darwin famously expounded upon how facial expressions serve as manifestations of emotional states in both human and diverse non-human species [6], however while he proposed a commonality across species for a given emotion, this finding has recently been challenged. Thus while mammals are known to produce facial expressions [7], the mechanistic rules governing this in relation to internal emotional states may vary between species. Consequently, facial expressions and their variability between species are getting increased interest as potential indicators of internal states in the domain of animal emotions and welfare research.

The golden standard for the objective evaluation of dynamics of facial expressions within the realm of human emotion research is the Facial Action Coding System—FACS [8, 9]. FACS has recently been adapted for different non-human species, including several non-human primates (e.g. orangutans [10], chimpanzees [11], macaques [12, 13]), marmosets [14], dogs [15], cats [16] and horses [17]. The latter are of particular interest due to their lateral eye placement accompanied by face elongation.

Horses are an understudied species in the context of emotion research. Being highly social animals, they have complex social frameworks [18, 19]. They have a well-developed communication through nuanced visual cues, including subtle shifts in eye direction, ear positioning, and facial expressions [17, 20]. Wathan et al. [21] presented evidence for their ability to distinguish between distinct facial expressions when presented with images of fellow horses, such as those conveying aggressive, positively attentive, or relaxed states.

As with many other species, facial expressions in horses have so far been investigated mainly in the context of pain. A number of grimace scales for assessing pain in horses, such as the Horse Grimace Scale (HGS) [22], Equine Pain Face [23] The Equine Utrecht University Scale for Facial Assessment of Pain (EQUUS-FAP) [24] and the FEReq instrument for ridden horses [25]. Merkies et al. [26] studied eye blink and eyelid twitches in relation to negative affective states. Hintze et al. [27] further focused on the eye area addressing eye wrinkles as a potential tool to evaluate emotional valence in horses, testing horses in different situations, two of them being food anticipation (positive emotional valence) and food competition inducing frustration (negative emotional valence). An important feature for separation between these positive and negative situations was found to be the change in the angle between the highest wrinkle and the line through the eyeball. Ears are another important facial parts studied in the context of affective states in horses and found to be of importance in fear [28], vigilance [29], as well as pain [22].

Ricci-Bonot and Mills [30] studied facial expressions in horses in a controlled experiment, testing n = 30 horses across three situations involving potential availability of food: one positive situation—anticipation of a reward, and two negative situations—frustration at waiting for a reward, and disappointment at the loss of the reward. Horse facial expressions were coded using the EquiFACS coding system. While the study could not identify facial markers to differentiate anticipation, significant difference was found in the occurrence of 9 actions and behaviors between the two negative situations. The action units ‘eye white increase’ (AD1), ‘ear rotator’ (EAD104), and ‘biting feeder’ were more likely in the frustration phase, while ‘blink’ (AU145), ‘nostril lift’ (AUH13), ‘tongue show’ (AD19), ‘chewing’ (AD81) and ‘licking feeder’ were more likely in the ‘disappointment’ phase.

Manual behavior analysis methods have many limitations, such as being prone to bias and error [31], as well as requiring rater agreement studies and extensive human training. Computer Vision based approaches provide an attractive alternative. Broome et al. [32] provides a comprehensive review of state-of-the-art approaches of this type in the context of affect recognition in animals.

As already indicated, the majority of these works focus on pain recognition, addressing species including rodents [33–35], sheep [36], and cats [37]. Several works have addressed automation of pain recognition in horses [38–40]. Lencioni et al. [38] presented a model, based on a Convolutional Neural Network (CNN), with an overall accuracy of 75.8% while classifying pain on three levels: not present, moderately present, and obviously present. While classifying between two categories (pain not present and pain present) the overall accuracy reached 88.3%. However, the validation method used did not leave one animal out, which is the golden standard in this context (see [32] for a discussion), and may have resulted in lower performance. This work used only input of single frames. Another study focusing on horse facial pain expressions was presented by Pessanha et al. [41]. The presented pipeline automatically determines the quantitative pose of the equine head and localizes facial landmarks, based on which classification is made. The manual scoring of pain was performed using the Equine Utrecht University Scale for Automated Recognition in Facial Assessment of Pain (EQUUS-ARFAP) [42]. This scale has not been validated, moreover a significant disagreement between scorers was reported. The pain prediction is done for each region of interest separately, models for some regions had a good performance in binary classification of pain/no pain (orbital tightening had F1 score of 0.86, ears 0.72), while the majority had lower performance. Hummel et al. [40] also focused on facial expressions for pain recognition in horses, presenting a hierarchical system for pose-specific automatic pain prediction on horse faces, exploring also its extension to donkeys. While 0.51–0.88 F1 score was achieved in pain recognition in horses, the transfer to donkeys was difficult. Another work on horse pain by Broome et al. addressed the whole body of the horse and used more sophisticated methods taking videos as input [39]. In follow-up studies, also transfer from acute to low grade orthopedic pain in horses was also addressed [43], as well as semi-supervised approaches with video [44].

To the best of our knowledge, only one study addressed so far emotional state recognition in horses. Corujo et al. [45] addressed some states including “alarmed”, “annoyed”, “curious”, and “relaxed”, defining each of them in terms of eyes, ears, nose and neck behavior. However, these definitions are not objective and nor operationally defined and so are open for observer interpretation (using descriptions such as ‘relaxed’), leading to low reliability of ground truth annotation.

The study presented here is the first to explore automated recognition of horse emotional states from facial expressions, using a dataset collected from a carefully designed experimental protocol of Ricci-Bonot and Mills [30]. In the protocol, similar to the one developed in [46] for dogs, the context defines the emotional states of horses, which were tested in three different scenarios involving the potential availability of food: anticipation of a reward, considered a positive emotional state; frustration at waiting for a reward and disappointment at the loss of the reward—both considered negative emotional states. Tests were conducted in a stable with a feeding device fixed outside the stable within reach of the horse. Analysis of video recordings of facial expressions of the horses was undertaken using the Horse Facial Action Coding System (EquiFACS), an objective system for coding facial movements on the basis of the contraction of underlying muscles, as well as their behaviors. This dataset creates a unique experimental environment for exploring different machine learning approaches in the context of emotion recognition. Specifically, we explore two routes to automated emotion recognition. The first approach uses deep learning, taking videos as input, and analyzing them frame by frame, then aggregating them for an emotional state prediction. The second approach takes as input the EquiFACS coding of the video and uses machine learning for making a prediction of an emotional state.

## Methods

### Dataset

The dataset used in this study was collected as part of a previous study by Ricci-Bonot and Mills [30]. The delegated authority of the University of Lincoln Research Ethics Committee approved this research (UoL2021_6910) and all methods were carried out in accordance with the University Research Ethics Policy and the ethical guidelines of ISAE [47]. Written informed consent was obtained from the owner of all horses used in the research. No further ethical approval was required for the current *in silico* work. All experiments were performed in accordance with relevant guidelines and regulations. The study is reported in accordance to ARRIVE guidelines.

A total of 30 videos were obtained from 31 horses involved in the experiment conducted by Ricci-Bonot et al. [30]. The horses belonged to different breeds, including Cob Normand, French saddle, Haflinger, Hungarian, Pinto cross Trotter, and some of unknown breed. The age range of the horses was 2 to 23 years, with an average age of 11.5 years and a standard deviation of 6.6. The experiment included 1 entire male, 10 geldings, and 20 females. One horse failed the training phase for food anticipation and all its videos were consequently excluded from the experiment.

The dataset included overall 296 video samples of 3-seconds length recorded at a frame rate of 60 frames per second, each frame resolution is 1920x1080 pixels.

Tests were conducted in a stable with a feeding device fixed outside the stable within reach of the horse using the protocol which is fully described in Ricci-Bonot and Mills [30]. Each subject was tested and recorded once in the baseline condition and three times on each of the anticipation, frustration and disappointments conditions, resulting in a data compound of 87 recordings of anticipation states, 30 recordings of baseline states, 90 recordings of disappointment states and 89 recordings of frustration states. Some videos cannot be used due to lack of enough visibility.

All the video samples were coded by a certified EquiFACS coder (C.R.B.) based on the EquiFACS manual. All action units, action descriptors and other variables were coded as present or absent; for the analysis, only EquiFACS variables shown to be reliable by a second EquiFACS coder and occurring in more than 10% of one of the four situations (baseline, anticipation, frustration and disappointment) were considered; In order to ensure the reliability of the coding, a second certified EquiFACS coder (N.J.) coded more than 10% of the video samples. Table 1 presents the EquiFacs variables that were eventually used in the analysis.

**Table 1:** EquiFACS variables (Action Units (AUs), Action Descriptors (ADs), Ear Action Descriptors (EADs) and additional variables) used in [30] after eliminating low frequency and low agreement variables.

Type	Code	Name	Describtion
AU	145	Blink	Both eyelids move towards each other to close the eye for less than 0.5 s.
AU	47	Half blink	Both eyelids move towards each other but the eye is not closed completely.
AD	1	Eye white increase	White sclera becomes visible in any part of the eye.
EAD	101	Ears forward	One or both ears are turned or swivel forward
EAD	103	Ear flattener	One or both ears are flattened and abducted.
EAD	104	Ear rotator	Ears are rotated laterally and caudally.
AU	10	Upper lip raiser	Central part of the upper lip raises straight up.
AU	12	Lip corner puller	Lip corners are pulled back caudally.
AU	16	Lower lip depressor	Lower lip pulls down ventrally
AU	18	Lip pucker	Upper lip protrude, being pushed forward
AU	25	Lips part	Lips separation
AU	26	Jaw drop	Lower jaw is lowered in a relaxed movement
AUH	13	Nostril lift	Nostril elongates
AD	19	Tongue show	Tongue is shown and it reaches beyond the teeth
AD	81	Chewing	Upper and lower jaw side-to-side grinding movement.
AD	51	Head turn left	Head moves left along a vertical axis.
AD	52	Head turn right	Head moves right along a vertical axis
AD	53	Head up	Head moves upwards
AD	54	Head down	Head moves downwards.
AD	56	Head tilt right	Head is titled to the right side.
VC	72	Lower face not visible	
Var		Licking feeder	Tongue extends through the teeth and the lips, and makes contact with the feeder.
Var		Biting the feeder	Horse takes a part of the feeder in its mouth by grabbing it between its teeth.

### AI pipelines overview

For narrative purposes we preface our results with essential and practical aspects to improve understanding for those less familiar with AI methods, presenting a high-level overview of the used approaches.

**Fig 1:**
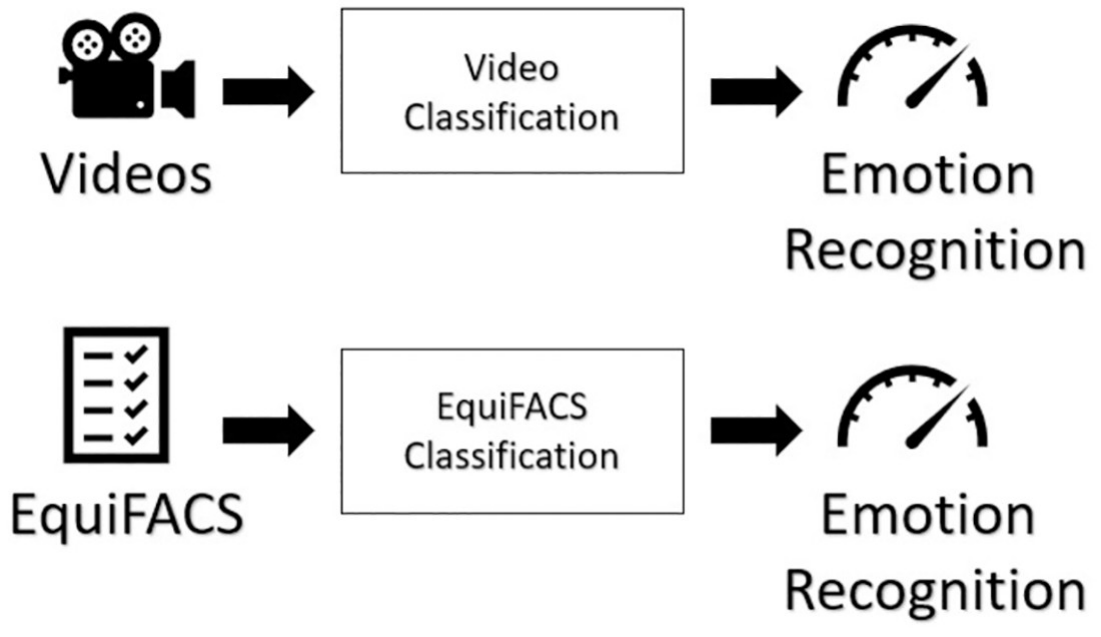
A high-level overview of the pipelines.

We compare the pain classification performance of two different pipelines utilizing two different types of input. The first pipeline takes as input 3sec long video recordings, the second takes as input the EquiFACS coding information. Fig 1 presents the two pipelines. S1 Appendix presents further technical details on the pipeline.

### Video classification pipeline

The pipeline for video classification used in this study follows the approach presented in [48], making a sophisticated use of the availability of video data in two ways: we integrate temporal information by using the Grayscale Short-Term stacking (GrayST) method [49] to encode movement between consecutive frames into one frame. In addition, we also apply a frame selection technique to better exploit the availability of video data and improve performance.

**Fig 2:**
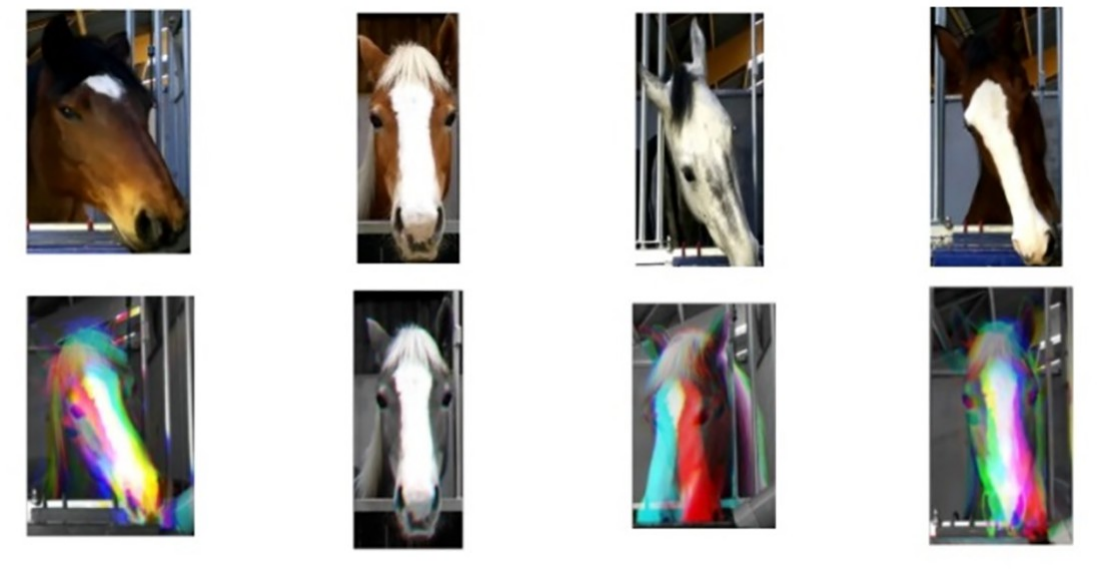
Examples of horse faces (top) and GrayST stacked frames. (bottom) for each of the emotional states from left to right: ‘Anticipation’, ‘Baseline’, ‘Disappointment’, ‘Frustration’.

The input to the model are videos. To remove background information, we crop the horse faces using Yolov5 object detection model [50]. Then we apply the GrayST method to incorporate temporal information for video classification without augmenting the computational burden. This sampling strategy involves substituting the conventional three color channels with three grayscale frames, obtained from three consecutive time steps. Consequently, the backbone network can capture short-term temporal dependencies while sacrificing the capability to analyze color. Fig 2 (top) displays examples of cropped horse faces (top) and GrayST stacked frames (bottom) for each of the emotional states from left to right: ‘Anticipation’, ‘Baseline’, ‘Disappointment’, ‘Frustration’. The bottom frames capture three consecutive frames: in the ‘Baseline’ case no movement of the horse is shown, while in the other three cases some movement is captured.

The next stage involves encoding each image into a 768-dimensional embedding vector employing a Visual Transformer (ViT [51]) trained in a self-supervised manner using DINO [52] with a batch size equals to 8. We extract the output of the final layer as a 768-dimensional embedding vector that will be used for emotion classification. Then, embedding vectors are fed to SVM models in a two-stage approach. Once the first SVM model is trained on all sampled frames, using the confidence levels (how confident the model is of its classification of a frame; confidence levels are computed as the probabilities of possible outcomes for samples in the dataset) to choose the top frames for each emotional class. Then the second SVM model is retrained using only the highest confidence frames. Fig 1 shows a high-level overview of the pipeline.

### EquiFACS classification pipeline

The EquiFACS data table for classification contains 296 rows (one for each video) and 25 columns: horse subject Id, emotional state and presence or absence of 23 different EquiFACS codes described above. The presence(absence) of a certain AU X in a specified video Y was marked as 1(0) on column of AU X and row of video Y. As part of the information was marked as not available, such entries were filled with 0.5. This data is then fed into a Decision Tree classifier. Fig 1 shows a high-level overview of the pipeline.

### Model performance

For measuring the performance of the models, we use standard evaluation metrics of accuracy, precision, recall and F1 (see, e.g., [37, 38] for further details). As a validation method [53], we use leave-one-subject-out cross validation with no subject overlap. Due to the relatively low numbers of horses (n = 30) in the dataset, following the stricter method is more appropriate [35, 39]. In our case this means that we repeatedly train on 29 subjects and test on the remaining subject. By separating the subjects used for training, validation and testing respectively, we enforce generalization to unseen subjects and ensure that no specific features of an individual are used for classification.

## Results

**Fig 3:**
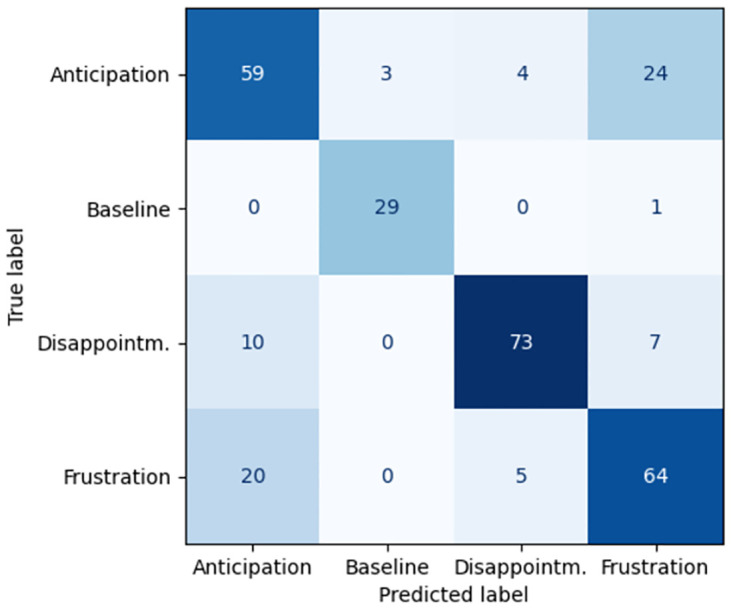
Confusion matrix for the video-based pipeline.

**Fig 4:**
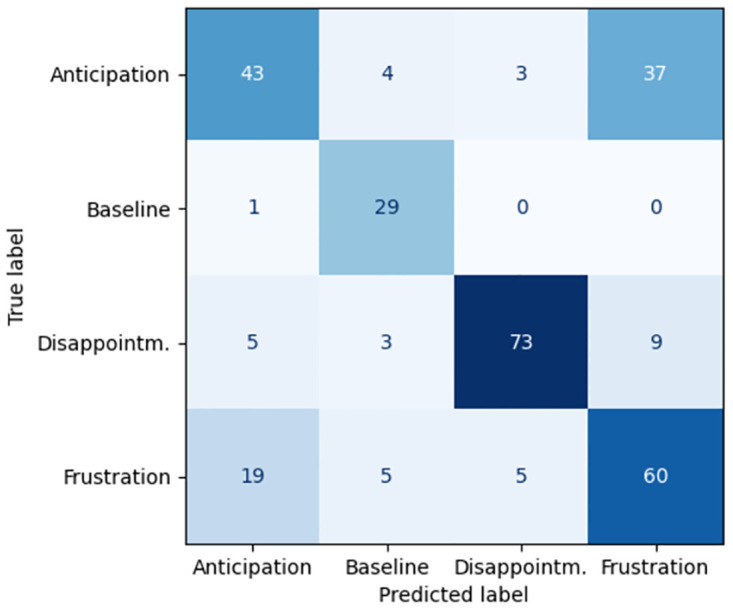
Confusion matrix for the Equifacs-based pipeline.

Table 2 presents our main results: the performance comparison between video-based pipeline and EquiFACS-based pipeline. We can see that the video-based pipeline outperforms the EquiFACS-based one, reaching 76% accuracy for separation between all the classes, as opposed to only 69% by the latter pipeline. It should be noted that this good performance was reached in a process of two phases, described in Table 3. The advantage of the EquiFACS-based classifier which performs lower is however its explainability in the form of a decision tree. Confusion matrices for the video-based and Equifacs-based pipelines can be found in Figs 3 and 4 respectively. It can be seen that separation between Anticipation and Frustration is difficult for both models. Thus Table 2 also presents classification performance for three states when Anticipation and Frustration are treated as one state, which greatly increases performance. The separation between the two ‘difficult’ states of Anticipation and Frustration reaches 61% accuracy in the video-based model, but had very low (46%) accuracy for te EquiFACS-based model.

**Table 2:** Performance of the two classifier pipelines using different configurations of classes: Separation into 4 classes, 3 classes unifying Anticipation and Frustration, and just 2 classes of Anticipation and Frustration.

	Video Classification				EquiFACS Classification			
Emotional States Configurations	Accuracy	Precision	Recall	F1	Accuracy	Precision	Recall	F1
4 Classes (Baseline/Anticipation/Disappointment/Frustration)	0.76	0.84	0.79	0.77	0.69	0.69	0.70	0.69
3 Classes (Baseline/Anticipation&Frustration/Disappointment)	0.90	0.93	0.90	0.90	0.88	0.88	0.89	0.88
2 Classes (Anticipation/Frustration)	0.61	0.65	0.60	0.56	0.46	0.46	0.46	0.46

**Table 3:** Video emotion classification results.

Model	Accuracy	Precision	Recall	F1
Face Cropping	0.67	0.78	0.71	0.69
Face Cropping + GrayST	0.68	0.77	0.71	0.70
Face Cropping + GrayST + k = 100	**0.76**	**0.84**	**0.79**	**0.77**

## Discussion

The present study is the first to explore automated recognition of horse emotional states focusing on diverse facial expressions, based on a carefully designed controlled experimental setup for dataset creation and annotation.

While facial muscular tone may decline with age [54] and facial morphology vary with factors like sex and breed, central to the idea of emotional expression is that reliable changes can be predicted regardless of these factors. Thus although factors like eye wrinkling might change with sex [55] and even be related to emotion, this expression cannot be used as a reliable general marker of emotion in horses, because of this difference. Therefore since we are interested in generic markers we do not attempt to model the effect of factors such as breed, gender or age into our models.

We presented classifier pipelines of two different types: deep learning video based and EquiFACS-based. The former reaches 76% accuracy in separating the four emotional states, while the latter has lower performance (69%). This could be an indication that EquiFACS contains less information that raw video, and there are subtle nuances not captures by the EquiFACS annotation system. This is further strengthened by the fact that the deep learning classifier outperforms EquiFACS also in separating the two “difficult” cases of Anticipation vs. Frustration, reaching 61% accuracy.

An EquiFACS-based approach was used in the original study [30], which involved observer based-coding, and this approach, even in an automated process, has one crucial benefit: explainability. As discussed in [37, 56], deep learning models have a ‘black-box’ nature, and it is important to understand *how* machines classify emotional states, exploring *explainability* (what is the rationale behind the machine’s decision?), and *interpretability* (how is the model structure related to making such decision?) [57]. These topics are fundamental in AI, and are addressed by a huge body of research [58, 59].

**Fig 5:**
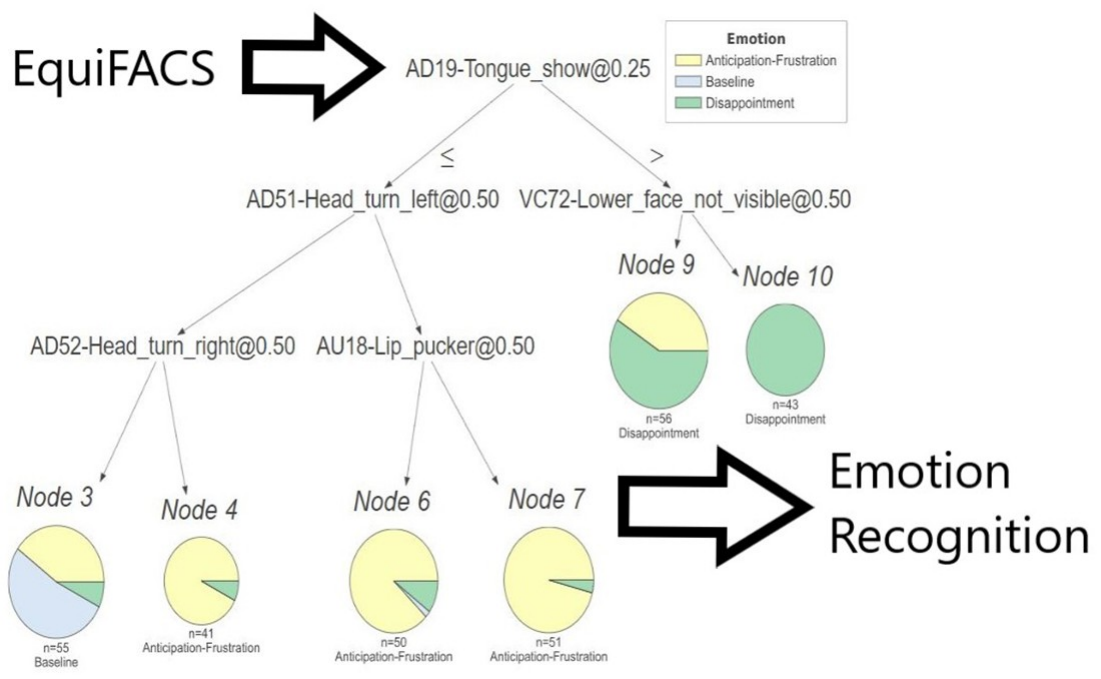
The EquiFACS-based Decision Tree.

The EquiFACS-based Decision Tree presented in this study allows us to answer such questions by observing the tree structure, represented by ‘if-then’ rules. From Fig 5 depicting the tree, one can imply, e.g., that the machine chooses “Baseline” when neither of the action units AD19-Tongueshow, AD-51-Head_turn_left nor AD-52-Head_turn_right are present (see the leftmost branch of the tree). When AD-19-Tongue_show is present, either when the lower face part is visible or not (the VC72-Lower_face_not_visible indicator is present or not), the machine chooses “Disappointment” (see the rightmost branch of tree). Otherwise, “Anticipation or Frustration” is derived. This suggests that the system is using more than facial expression, but the wider movement of the head as part of the classification process. The risk of this being artefactual, arising from the design of the study (based on a food delivery system) needs to be carefully considered, and thus generalization about emotional state made with care. A similar phenomenon could arise within the generally superior deep learning video based approach, but we have no way of knowing this. Thus replication studies examining these emotions in horses in other contexts are essential and will strengthen the database used for deriving solid conclusions.

For a possible explanation of why the states of anticipation and frustration could not be well separated in our study, despite the fact that they had a stronger separation in the study of dogs [46], we refer the reader to Ricci-Bonot and Mills [30]. Whilst the authors considered it possible that there was a lack of facial differences between positive anticipation and frustration in horses or that feeding in the context of the experiment is a largely frustrating event, it was also thought that it might be an artefact of using a 1–0 sampling method, which meant the detail within a video may not have been captured [30]. The fact that the video-based deep learning model has 61% accuracy in this case indicates that some visual signal is there, and the explainability of this classifier should be explored in future research, with future experimental protocol designs aimed at better separation of these two emotional states.

## Supporting information

S1 Appendix Technical details on the video-based classifier. (PDF)

## References

[1] Sneddon LU, Elwood RW, Adamo SA, Leach MC. Defining and assessing animal pain. Animal Behaviour. 2014;97:201–212. doi: 10.1016/j.anbehav.2014.09.007

[2] de Vere AJ, Kuczaj SA. Where are we in the study of animal emotions? Wiley Interdisciplinary Reviews: Cognitive Science. 2016;7(5):354–362. 27327075 10.1002/wcs.1399

[3] Taylor K, Mills D. Is quality of life a useful concept for companion animals? Animal welfare. 2007;16(S1):55–65. doi: 10.1017/S0962728600031730

[4] Duncan IJ. Animal welfare defined in terms of feelings. Acta Agriculturae Scandinavica Section A Animal Science Supplementum (Denmark). 1996;.

[5] Boissy A, Arnould C, Chaillou E, Désiré L, Duvaux-Ponter C, Greiveldinger L, et al. Emotions and cognition: a new approach to animal welfare. Animal Welfare. 2007;16(2):37–43. doi: 10.1017/S0962728600031717

[6] Darwin C. The expression of emotions in animals and man. London: Murray. 1872;11:1872.

[7] Diogo R, Abdala V, Lonergan N, Wood B. From fish to modern humans–comparative anatomy, homologies and evolution of the head and neck musculature. Journal of Anatomy. 2008;213(4):391–424. doi: 10.1111/j.1469-7580.2008.00953.x 18657257 PMC2644766

[8] Ekman P, Friesen WV. Facial Action Coding System: Manual. Palo Alto, Calif: Consulting Psychologists Press; 1978.

[9] Ekman P, Friesen W. Facial action coding system: a technique for the measurement of facial movement. Environmental Psychology & Nonverbal Behavior. 1978;.

[10] Caeiro C, Waller B, Zimmerman E, Burrows A, Davila Ross M. OrangFACS: A muscle-based movement coding system for facial communication in Orangutans. International Journal of Primatology. 2013;34:115–129. doi: 10.1007/s10764-012-9652-x

[11] Parr LA, Waller BM, Vick SJ, Bard KA. Classifying chimpanzee facial expressions using muscle action. Emotion. 2007;7(1):172. doi: 10.1037/1528-3542.7.1.172 17352572 PMC2826116

[12] Clark PR, Waller BM, Burrows AM, Julle-Danière E, Agil M, Engelhardt A, et al. Morphological variants of silent bared-teeth displays have different social interaction outcomes in crested macaques (Macaca nigra). American Journal of Physical Anthropology. 2020;173(3):411–422. doi: 10.1002/ajpa.24129 32820559

[13] Correia-Caeiro C, Holmes K, Miyabe-Nishiwaki T. Extending the MaqFACS to measure facial movement in Japanese macaques (Macaca fuscata) reveals a wide repertoire potential. PLOS ONE. 2021;16(1):e0245117. doi: 10.1371/journal.pone.0245117 33411716 PMC7790396

[14] Correia-Caeiro C, Burrows A, Wilson DA, Abdelrahman A, Miyabe-Nishiwaki T. CalliFACS: The common marmoset Facial Action Coding System. PloS one. 2022;17(5):e0266442. doi: 10.1371/journal.pone.0266442 35580128 PMC9113598

[15] Waller B, Caeiro C, Peirce K, Burrows A, Kaminski J, et al. DogFACS: the dog facial action coding system. Manual University of Portsmouth. 2013;.

[16] Caeiro CC, Burrows AM, Waller BM. Development and application of CatFACS: Are human cat adopters influenced by cat facial expressions? Applied Animal Behaviour Science. 2017;. doi: 10.1016/j.applanim.2017.01.005

[17] Wathan J, Burrows AM, Waller BM, McComb K. EquiFACS: the equine facial action coding system. PLOS ONE. 2015;10(8):e0131738. doi: 10.1371/journal.pone.0131738 26244573 PMC4526551

[18] Feh C. Relationships and communication in socially natural horse herds. The domestic horse. 2005; p. 83–93.

[19] Cozzi A, Sighieri C, Gazzano A, Nicol CJ, Baragli P. Post-conflict friendly reunion in a permanent group of horses (Equus caballus). Behavioural processes. 2010;85(2):185–190. doi: 10.1016/j.beproc.2010.07.007 20659538

[20] Wathan J, McComb K. The eyes and ears are visual indicators of attention in domestic horses. Current Biology. 2014;24(15):R677–R679. doi: 10.1016/j.cub.2014.06.023 25093554 PMC4123162

[21] Wathan J, Proops L, Grounds K, Mccomb K. Horses discriminate between facial expressions of conspecifics. Scientific reports. 2016;6(1):38322. doi: 10.1038/srep38322 27995958 PMC5171796

[22] Dalla Costa E, Minero M, Lebelt D, Stucke D, Canali E, Leach MC. Development of the Horse Grimace Scale (HGS) as a pain assessment tool in horses undergoing routine castration. PLOS ONE. 2014;9(3):e92281. doi: 10.1371/journal.pone.0092281 24647606 PMC3960217

[23] Gleerup KB, Forkman B, Lindegaard C, Andersen PH. An equine pain face. Veterinary anaesthesia and analgesia. 2015;42(1):103–114. doi: 10.1111/vaa.12212 25082060 PMC4312484

[24] van Loon JP, Van Dierendonck MC. Monitoring acute equine visceral pain with the Equine Utrecht University Scale for Composite Pain Assessment (EQUUS-COMPASS) and the Equine Utrecht University Scale for Facial Assessment of Pain (EQUUS-FAP): a scale-construction study. The Veterinary Journal. 2015;206(3):356–364. doi: 10.1016/j.tvjl.2015.08.023 26526526

[25] Mullard J, Berger JM, Ellis AD, Dyson S. Development of an ethogram to describe facial expressions in ridden horses (FEReq). Journal of Veterinary Behavior. 2017;18:7–12. doi: 10.1016/j.jveb.2016.11.005

[26] Merkies K, Ready C, Farkas L, Hodder A. Eye blink rates and eyelid twitches as a non-invasive measure of stress in the domestic horse. Animals. 2019;9(8):562. doi: 10.3390/ani9080562 31443315 PMC6721043

[27] Hintze S, Smith S, Patt A, Bachmann I, Würbel H. Are eyes a mirror of the soul? What eye wrinkles reveal about a horse’s emotional state. PLOS ONE. 2016;11(10):e0164017. doi: 10.1371/journal.pone.0164017 27732647 PMC5061373

[28] Leiner L, Fendt M. Behavioural fear and heart rate responses of horses after exposure to novel objects: Effects of habituation. Applied Animal Behaviour Science. 2011;131(3-4):104–109. doi: 10.1016/j.applanim.2011.02.004

[29] Hausberger M, Fureix C, Lesimple C. Detecting horses’ sickness: In search of visible signs. Applied Animal Behaviour Science. 2016;175:41–49. doi: 10.1016/j.applanim.2015.09.005

[30] Ricci-Bonot C, Mills DS. Recognising the facial expression of frustration in the horse during feeding period. Applied Animal Behaviour Science. 2023;265:105966. doi: 10.1016/j.applanim.2023.105966

[31] Anderson DJ, Perona P. Toward a science of computational ethology. Neuron. 2014;84(1):18–31. doi: 10.1016/j.neuron.2014.09.005 25277452

[32] Broomé S, Feighelstein M, Zamansky A, Lencioni GC, Andersen PH, Pessanha F, et al. Going Deeper than Tracking: a Survey of Computer-Vision Based Recognition of Animal Pain and Affective States. arXiv preprint arXiv:220608405. 2022;.

[33] Sotocina SG, Sorge RE, Zaloum A, Tuttle AH, Martin LJ, Wieskopf JS, et al. The Rat Grimace Scale: a partially automated method for quantifying pain in the laboratory rat via facial expressions. Molecular pain. 2011;7:1744–8069. doi: 10.1186/1744-8069-7-55 PMC3163602 21801409

[34] Tuttle AH, Molinaro MJ, Jethwa JF, Sotocinal SG, Prieto JC, Styner MA, et al. A deep neural network to assess spontaneous pain from mouse facial expressions. Molecular pain. 2018;14:1744806918763658. doi: 10.1177/1744806918763658 29546805 PMC5858615

[35] Andresen N, Wöllhaf M, Hohlbaum K, Lewejohann L, Hellwich O, Thöne-Reineke C, et al. Towards a fully automated surveillance of well-being status in laboratory mice using deep learning: Starting with facial expression analysis. PLOS ONE. 2020;15(4):e0228059. doi: 10.1371/journal.pone.0228059 32294094 PMC7159220

[36] Mahmoud M, Lu Y, Hou X, McLennan K, Robinson P. Estimation of pain in sheep using computer vision. In: Moore RJ, editor. Handbook of Pain and Palliative Care. Cham: Springer; 2018. p. 145–157.

[37] Feighelstein M, Shimshoni I, Finka LR, Luna SP, Mills DS, Zamansky A. Automated recognition of pain in cats. Scientific Reports. 2022;12(1):1–10. doi: 10.1038/s41598-022-13348-1 35688852 PMC9187730

[38] Lencioni GC, de Sousa RV, de Souza Sardinha EJ, Corrêa RR, Zanella AJ. Pain assessment in horses using automatic facial expression recognition through deep learning-based modeling. PLOS ONE. 2021;16(10):e0258672. doi: 10.1371/journal.pone.0258672 34665834 PMC8525760

[39] Broomé S, Gleerup KB, Andersen PH, Kjellstrom H. Dynamics are important for the recognition of equine pain in video. In: Proceedings of the IEEE/CVF Conference on Computer Vision and Pattern Recognition; 2019. p. 12667–12676.

[40] Hummel HI, Pessanha F, Salah AA, van Loon TJ, Veltkamp RC. Automatic pain detection on horse and donkey faces. In: 2020 15th IEEE International Conference on Automatic Face and Gesture Recognition (FG 2020). IEEE; 2020. p. 793–800.

[41] Pessanha F, Salah AA, van Loon T, Veltkamp R. Facial image-based automatic assessment of equine pain. IEEE Transactions on Affective Computing. 2022;.

[42] Jones A. Development and validation of a dog personality questionnaire (Doctoral dissertation). University of Texas at Austin, TX. 2008;.

[43] Broomé S, Ask K, Rashid-Engström M, Haubro Andersen P, Kjellström H. Sharing pain: Using pain domain transfer for video recognition of low grade orthopedic pain in horses. PloS one. 2022;17(3):e0263854. doi: 10.1371/journal.pone.0263854 35245288 PMC8896717

[44] Rashid M, Broomé S, Ask K, Hernlund E, Andersen PH, Kjellström H, et al. Equine pain behavior classification via self-supervised disentangled pose representation. In: Proceedings of the IEEE/CVF Winter Conference on Applications of Computer Vision; 2022. p. 1646–1656.

[45] Corujo LA, Kieson E, Schloesser T, Gloor PA. Emotion Recognition in Horses with Convolutional Neural Networks. Future Internet. 2021;13(10):250. doi: 10.3390/fi13100250

[46] Bremhorst A, Sutter NA, Würbel H, Mills DS, Riemer S. Differences in facial expressions during positive anticipation and frustration in dogs awaiting a reward. Scientific reports. 2019;9(1):1–13. doi: 10.1038/s41598-019-55714-6 31848389 PMC6917793

[47] Sherwin CM, Christiansen SB, Duncan IJ, Erhard HW, Lay DC Jr, Mench JA, et al. Guidelines for the ethical use of animals in applied ethology studies. Applied Animal Behaviour Science. 2003;81(3):291–305. doi: 10.1016/S0168-1591(02)00288-5

[48] Feighelstein M, Ehrlich Y, Naftaly L, Alpin M, Nadir S, Shimshoni I, et al. Deep learning for video-based automated pain recognition in rabbits. Scientific Reports. 2023;13(1):14679. doi: 10.1038/s41598-023-41774-2 37674052 PMC10482887

[49] Kim K, Gowda SN, Mac Aodha O, Sevilla-Lara L. Capturing temporal information in a single frame: Channel sampling strategies for action recognition. arXiv preprint arXiv:220110394. 2022;.

[50] Zhu X, Lyu S, Wang X, Zhao Q. TPH-YOLOv5: Improved YOLOv5 Based on Transformer Prediction Head for Object Detection on Drone-captured Scenarios; 2021.

[51] Dosovitskiy A, Beyer L, Kolesnikov A, Weissenborn D, Zhai X, Unterthiner T, et al. An image is worth 16x16 words: Transformers for image recognition at scale. arXiv preprint arXiv:201011929. 2020;.

[52] Caron M, Touvron H, Misra I, Jégou H, Mairal J, Bojanowski P, et al. Emerging Properties in Self-Supervised Vision Transformers. In: Proceedings of the IEEE/CVF International Conference on Computer Vision; 2021.

[53] Refaeilzadeh P, Tang L, Liu H. In: LIU L, ÖZSU MT, editors. Cross-Validation. Boston, MA: Springer US; 2009. p. 532–538. Available from: 10.1007/978-0-387-39940-9_565.

[54] Cotofana S, Assemi-Kabir S, Mardini S, Giunta RE, Gotkin RH, Moellhoff N, et al. Understanding facial muscle aging: a surface electromyography study. Aesthetic Surgery Journal. 2021;41(9):NP1208–NP1217. doi: 10.1093/asj/sjab202 33942051

[55] Schanz L, Krueger K, Hintze S. Sex and age don’t matter, but breed type does—Factors influencing eye wrinkle expression in horses. Frontiers in Veterinary Science. 2019;6:154. doi: 10.3389/fvets.2019.00154 31192235 PMC6549476

[56] Boneh-Shitrit T, Feighelstein M, Bremhorst A, Amir S, Distelfeld T, Dassa Y, et al. Explainable automated recognition of emotional states from canine facial expressions: the case of positive anticipation and frustration. Scientific reports. 2022;12(1):22611. doi: 10.1038/s41598-022-27079-w 36585439 PMC9803655

[57] Escalante HJ, Guyon I, Escalera S, Jacques J, Madadi M, Baró X, et al. Design of an explainable machine learning challenge for video interviews. In: 2017 International Joint Conference on Neural Networks (IJCNN); 2017. p. 3688–3695.

[58] Linardatos P, Papastefanopoulos V, Kotsiantis S. Explainable ai: A review of machine learning interpretability methods. Entropy. 2020;23(1):18. doi: 10.3390/e23010018 33375658 PMC7824368

[59] Gilpin LH, Bau D, Yuan BZ, Bajwa A, Specter M, Kagal L. Explaining explanations: An overview of interpretability of machine learning. In: 2018 IEEE 5th International Conference on data science and advanced analytics (DSAA). IEEE; 2018. p. 80–89.

