# Correction: Automated recognition of emotional states of horses from facial expressions

**DOI:** 10.1371/journal.pone.0319501

**Published:** 2025-02-12

**Authors:** Marcelo Feighelstein, Claire Ricci-Bonot, Hana Hasan, Hallel Weinberg, Tidhar Rettig, Maya Segal, Tomer Distelfeld, Ilan Shimshoni, Daniel S. Mills, Anna Zamansky

The second author’s name is spelled incorrectly. The correct name is: Claire Ricci-Bonot. The correct citation is: Feighelstein M, Ricci-Bonot C, Hasan H, Weinberg H, Rettig T, Segal M, et al. (2024) Automated recognition of emotional states of horses from facial expressions. PLOS ONE 19(7): e0302893. https://doi.org/10.1371/journal.pone.0302893.

There are errors in the author affiliations. The correct affiliations are as follows:

Marcelo Feighelstein^1^, Claire Ricci-Bonot^2^, Hana Hasan^3^, Hallel Weinberg^3^, Tidhar Rettig^3^, Maya Segal^4^, Tomer Distelfeld^4^, Ilan Shimshoni^1^, Daniel S. Mills^2^, and Anna Zamansky^1^

**1** Information Systems Department, University of Haifa, Haifa, Israel, **2** Department of Life Sciences, Joseph Banks Laboratories, University of Lincoln, Lincoln, United Kingdom, **3** Computer Science Department, University of Haifa, Haifa, Israel, **4** Faculty of Electrical Engineering, Technion, Israel Institute of Technology, Haifa, Israel.

The third author Hana Hasan, should also be noted as first co-author on this work.
