# Supplementary material for: Automated recognition of emotional states of horses from facial expressions
Source: PLoS One. 2024 Jul 15;19(7):e0302893. doi: 10.1371/journal.pone.0302893 (PMC11249218; doi:10.1371/journal.pone.0302893)
Supplement: S1 Appendix — (PDF) [file pone.0302893.s001.pdf]

Appendix

Video-based Classifier Details

Table 3 displays the performance outcomes of the pipeline performing the following two phases.

1. Naive phase. The initial SVM model referred to as ”Model 1” was trained first on all frames' embedding vectors and did not utilize GrayST pre-processing. The SVM model achieved an accuracy of 65% employing Face Cropping. However, when GrayST pre-processing was employed, the model's performance slightly improved to 66%. The SVM model was trained with regularization parameter C equals to 1, radial basis function (RBF) kernel and kernel coefficient gamma equals to 1 / (number of features = 768 * variance of full table of embedding vectors). In order to emulate a balanced dataset, we adjust the weights assigned to each class in inverse proportion to their frequencies in the input data.

2. Improved phase. The next ”Model 2”, on the other hand, was only trained on the embedding vectors of frames with the highest confidence (obtained in ”Model 1”), achieving an improved accuracy of 76%. Note that to test ”Model 2” we use the same strict cross-validation method of leave-one-subject-out to avoid over-fitting. ”Model 2” is tested on embedding vectors of all frames of videos belonging to horses taken out for testing. The same parameters of Naive Phase were used for training of the SVM model on this phase.

The decisions of the chosen emotional state per frame are then aggregated using majority voting, and the classification decision per video is reached.

The aggregated results are presented in Table 3, which displays the video classification results using combinations of training sets consisting of all cropped and sampled frames, using or not using the GrayST aggregation method, and training only using High Confidence (k=100) frames or not.

The GrayST mechanism is used in the Naive phase to capture temporal information staking three consecutive frames converted to gray color into a single frame. As examples, the bottom frames on Figure 2 capture three consecutive frames. In the 'Baseline' case no movement of the horse is shown, while in the other three cases movement is captured.
